# Switching Dielectric Constant Near Room Temperature in a Molecular Crystal

**DOI:** 10.1002/advs.201500029

**Published:** 2015-04-14

**Authors:** Xiu‐Dan Shao, Xi Zhang, Chao Shi, Ye‐Feng Yao, Wen Zhang

**Affiliations:** ^1^ Ordered Matter Science Research CenterSoutheast UniversityNanjing211189JiangsuChina; ^2^ Department of Physics and Shanghai Key Laboratory of Magnetic ResonanceEast China Normal UniversityNorth Zhongshan Road 3663Shanghai200062China

**Keywords:** dielectric constants, molecular crystals, phase transitions, rotational dynamics, switchable materials

## Abstract

**The organic salt bis(2‐chloroethyl)amine hydrochloride** shows a sharp switching of its dielectric constant at 320 K. The switching property originates from the dynamic changes of the (2‐chloroethyl)ammonium cation between frozen and motional states, corresponding to a structural phase transition.

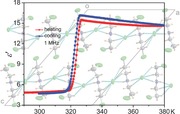

Switchable materials have been attracting great attention because of their potential applications as switches, sensors, and memory devices.^1^, ^2^, ^3^, ^4^ Upon external stimuli, these materials can show switch‐like behaviors between two (or more) states. Taking magnetic materials for example, spin crossover compounds can be switched between high‐ and low‐spin states via spin unparing and paring (e.g., t_2g_^4^e_g_^2^ ↔ t_2g_^6^e_g_^0^ in Fe(II) complexes) triggered by temperature, pressure, light, and/or chemicals.^3^, ^4^, ^5^, ^6^ As an electrical counterpart, switchable dielectric materials have only recently been identified.^7^, ^8^ They distinguish themselves by undergoing dielectric transitions between high‐ and low‐dielectric states, a switching property that looks like spin crossover, although the origin arises from atomic or ionic rearrangements rather than the spin pairing and unparing in spin crossover compounds.^7^, ^8^, ^9^, ^10^, ^11^ These types of materials enlarge the family of the widely used dielectric materials and would find potential applications in electrical and electronic industries.

The principle of switchable dielectric constant is straightforward. Microscopically, dielectric response of a molecular compound has three intrinsic origins, i.e., electronic polarization, ionic displacement, and dipolar reorientation.^12^ The last one, when it exists, makes a major contribution to the dielectric constant, corresponding to the high‐dielectric state. If the reorientation is frozen below a certain temperature, e.g., phase transition point (*T*_c_), the low‐dielectric state occurs. Thus, the switchable dielectric constant is realized by the motional changes of polar molecules or ions between motional and frozen states.

To find a switchable dielectric material is experimentally nontrivial because of little knowledge on the microscopically structural origin of the dielectric transition in a single‐phase material. A promising library of switchable dielectrics is the so‐called amphidynamic crystals^13^, ^14^ which consist of both mobile and static parts in the crystal lattices, such as dynamic porous frameworks^15^, ^16^, ^17^, ^18^ and molecular rotors.^19^, ^20^ In these types of compounds, the dynamics of the mobile polar components or guest molecules or ions can be fairly tuned by molecular design. However, requirements for a good switchable dielectric material are not easy to satisfy simultaneously in a compound, including step‐like sharp transition, proper working temperature, frequency independence, easy preparation, cheapness, processibility, and so on.^7^, ^8^, ^9^, ^10^, ^11^ Herein, we report an organic salt bis(2‐chloroethyl)amine hydrochloride (**1**), showing a sharp switching between high‐ and low‐dielectric states at 320 K. Detailed single‐crystal structural analysis and solid‐state NMR study reveal that the switchable dielectric constant is closely related to the changes of the dynamics of the bis(2‐chloroethyl)ammonium (BCEA) cations between the frozen and motional states in the structure. The compound is simple, commercially available, and easily processible, and shows a sharp dielectric switch just above room temperature. These advantages make it a good candidate for applications as a molecule‐based switchable material.

Compound **1** is a colorless crystalline compound. A structural phase transition was verified by differential scanning calorimetry (DSC) measurement (**Figure** **1**a). During a heating and cooling cycle in the temperature range 173−373 K, a pair of exo‐ and endo‐thermal peaks appears at 311 and 330 K, respectively. For convenience, the two phases are designated as room‐temperature phase (RTP) and high‐temperature phase (HTP). The pair of peaks centered at around 320 K is sharp. Enthalpy change around the *T*_c_ is then extracted from the heat flow curve with a value of 3.789 kJ mol^−1^ and the corresponding entropy change Δ*S* is calculated to be 11.84 J mol^−1^ K^−1^. Thus, an extent of disorderness in the HTP is estimated by Boltzmann equation Δ*S* = *R*ln(*N*) where *R* is the gas constant and *N* the ratio of the numbers of respective geometrically distinguishable orientations in the two phases to give the value of *N* of 4.154, suggesting a high disorderness in the HTP.

**Figure 1:**
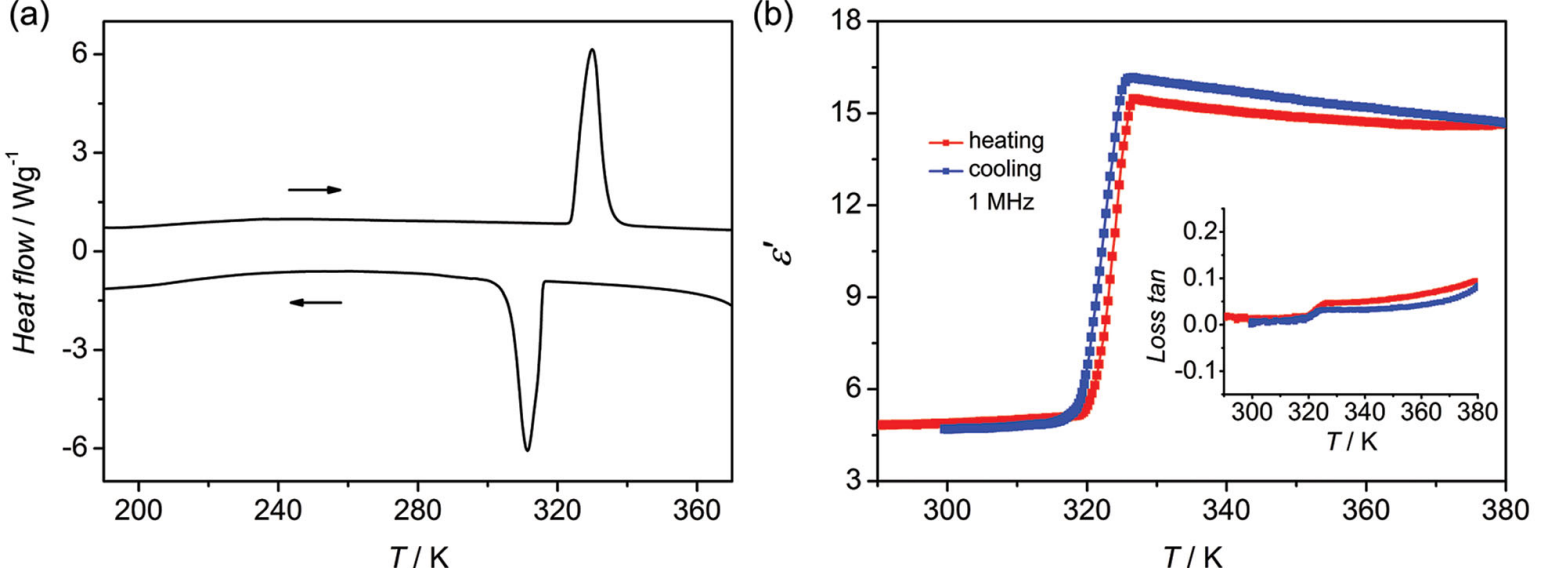
a) DSC curves of **1** measured in a heating‐cooling cycle. b) Temperature dependence of the real part of dielectric constant and loss tangent of **1** at 1 MHz.

Variable‐temperature (VT) dielectric spectra were measured on the power pressed pellet of **1** (Figure 1b and Figure S1, Supporting Information). The dielectric response (the real part *ε*′ of complex permittivity *ε* = *ε*′ − *iε*″) shows a sharp switching between low‐ and high‐dielectric states at 320 K. At 1 MHz and upon heating, the *ε*′ keeps stable at about 5 below 320 K, indicating a static or ordered phase of the sample. Then it jumps to 15.6 at 326 K, equivalent to a threefold increase. Above 326 K the *ε*′ keeps a little decrease with the increase of temperature and reaches a value of 14.6, reflecting a competition between polarization and thermal fluctuation disturbance.^12^ As to the dielectric loss, there is a small change around the *T*_c_. Little frequency dependence is observed in the measured frequency range 5−1000 kHz, indicating much faster polar motions (Figure S1, Supporting Information) than 1 MHz. It is known that the *ε*′ of a molecular material reflects thermally activated molecular rotations (disordered phase) and structural changes under an external electric field. The observed step‐like dielectric response in **1** indicates the occurrence of an order–disorder phase transition between the RTP and HTP at 320 K.

Structure–property relationship and dynamic characteristics of **1** are studied first by a crystal structure analysis. At 293 K (RTP), **1** crystallizes in the monoclinic space group *P*2/*c* with *a* = 15.29(2) Å, *b* = 5.057(6) Å, *c* = 22.19(2) Å, *β* = 130.86(5)°, and *V* = 1298(3) Å^3^.^21^ The basic structural unit is composed of one and a half pairs of BCEA cations and Cl ions (**Figure** **2**a). The cations show two different conformations, designated as A and B with a molar ratio of 2:1, that is, four A and two B cations in the unit cell. In the A cation, the terminal Cl1 and Cl2 atoms adopt gauche (61.81°) and anti (179.02°) forms to the N1 atom, respectively. In contrast, the terminal Cl3 atom has a gauche (66.11°) orientation to the N2 atom in the B cation. The different conformations in A and B reflect the significant packing effect of the crystal lattice on the cations (Figure S2, Supporting Information). This effect was also observed in the ^13^C NMR spectroscopy of the sample as discussed below.

**Figure 2:**
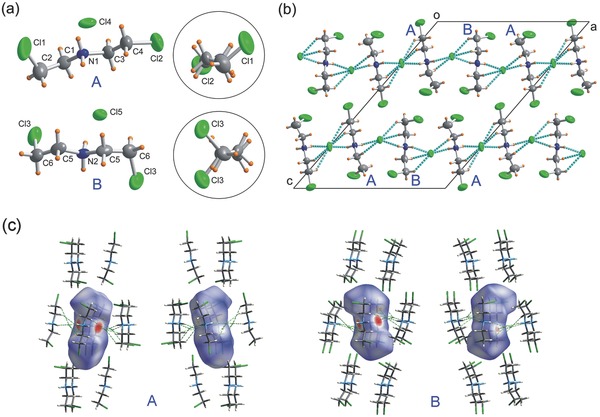
a) Side and top views of two conformations of BCEA (A and B) in the structural unit of **1** at 293 K. b) Hydrogen bonds in the crystal packing shown as dotted lines. c) Front and back views of Hirshfeld isosurfaces (above) of **1** at 293 K, indicating the relative strength of these intermolecular interactions surrounding the A and B cations. The red, white, and blue regions represent molecular contacts shorter than, equal to, and longer than the van der Waals distance (isovalue = 0.002 au), respectively.

Hydrogen bonds exist between the Cl anions and the H atoms, forming infinite 2D hydrogen‐bonding networks parallel to the *c* plane (Figure 2b). However, the terminal Cl atoms in the cations are not involved in these interactions and even in short contacts. A direct visualization of the distribution of the intermolecular interactions is gained by a Hirshfeld surface analysis.^22^, ^23^, ^24^, ^25^ It is found that the main interactions exist among the NH groups in the BCEA cations and Cl ions around them, shown as red areas in Figure 2c. However, the predominant white and blue areas of the surfaces indicate a loose‐packed nature of the cations in the crystal lattice. The difference of the intermolecular interactions between the A and B cations is clearly shown in the 2D fingerprint plots (Figure S3, Supporting Information). With the increase of the temperature from 113 to 313 K, the environment of the A cation obviously changes with the increase of the [H···Cl] interactions from 63.3% to 64.9% and the decrease of the [H···H] interactions from 33.5% to 32.2% while the environment of the B cation keeps stable, reflecting the strain‐releasing process of the A cation (Figure S4, Supporting Information). Temperature‐dependent cell parameters show a quicker change of the void than the cell volume, which may leave a room for the disorder of the cations to result in the phase transition at 320 K (Figure S5, Supporting Information).

Attempts to solve the crystal structure of **1** in the HTP were unsuccessful because of much weaker diffraction points than the RTP diffraction pattern. Fortunately, the cell parameters were obtained as *a* = 7.2698(13) Å, *b* = 7.2684(7) Å, *c* = 9.043(2) Å, *β* = 113.63(3)°, and *V* = 437.80(14) Å^3^ with a monoclinic space group *C*2/*m*. Compared with the data of the RTP, it is found the cell volume of HTP becomes one third of the RTP. So there are only two BCEA cations in the unit cell. This result indicates the A and B conformers become the same in the HTP most probably because of the easy transition caused by thermal agitation. Furthermore, VT powder X‐ray diffraction patterns of **1** were collected between 298−353 K (Figure S6, Supporting Information). The RTP and HTP patterns differ from each other greatly, indicating an obvious structural change.

VT IR/Raman spectra of **1** show striking differences between the RTP and HTP (Figures S7 and S8, Supporting Information). Single broad bands appear in the HTP instead of a splitting of the sharp ones in the RTP. This is an evidence of a local symmetry change of the cations. The two bands centered at 675 and 755 cm^−1^ are assigned to the C—Cl stretching vibrations in the gauche and anti forms, respectively.^26^ The former one that splits in the RTP is ascribed to the different environments of A and B cations. Clearly, they become the same in the HTP since the peaks merge to single ones. Furthermore, the change of the peak intensities indicates the proportion of the anti form increases in the HTP. The —NH_2_ vibration being clear at 3165 cm^−1^ in the IR spectra at 313 K nearly flattens at 333 K, reflecting a motion of the group in the crystal lattice.

The changes in the structure and dynamics of **1** from the RTP to HTP were further investigated by high resolution ^13^C NMR spectroscopy.^27^ Six peaks are well resolved in the spectrum of the sample below the *T*_c_ (**Figure** **3**a). The three low‐field signals are tentatively assigned to the CH_2_ groups and the three high‐field signals to the CH_2_Cl groups. The complete signal assignment, i.e., assigning the signals to the groups in a specific conformer of BCEA, is the subject of ongoing study. It is noted that the six signals in the spectrum are a good reflection of the two conformations of BCEA cations in the crystal lattice (Figure 1). Conformer B has a symmetric environment, and thus the CH_2_ and CH_2_Cl groups are expected to give rise to only two signals while A has an asymmetric environment and thus the two CH_2_ and CH_2_Cl groups give rise to four signals. The appearance of the total six signals in the ^13^C NMR spectrum is thus in line with the observation in the X‐ray diffraction. Above the *T*_c_, only two peaks are observed, indicating that the BCEA cations in the HTP are present in a uniform and high‐symmetric environment. Intriguingly, the static powder lineshapes of the ^13^C chemical shift anisotropy of CH_2_ and CH_2_Cl groups both show typical features of an axially symmetric tensor (Figure 3b). This indicates that above the *T*_c_ both of the groups likely undergo a restricted anisotropic motion close to a locally axial rotation. Further analysis indicates that the whole BCEA cations likely rotate along the long axis above the *T*_c_ (Figure S12, Supporting Information), which is considered to be of relevance to the switching of the dielectric constant below and above the *T*_c_. But, determination of the exact rotation axis via ^13^C chemical shift anisotropy (CSA) analysis is limited by lacking of the detailed information about the ^13^C CSA tensors (i.e., the three principal values and the tensor orientation in the molecular frame).

**Figure 3:**
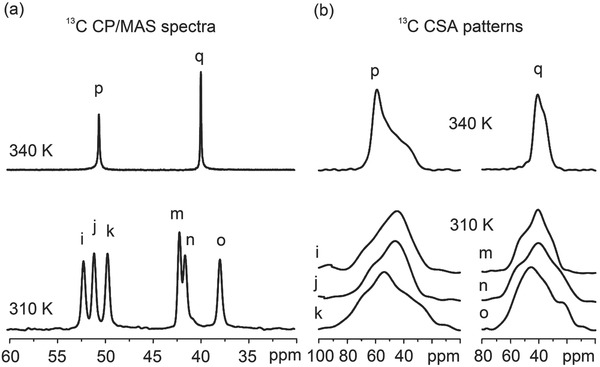
a) Solid‐state ^13^C NMR spectra of **1** below and above the *T*_c_. b) ^13^C CSA patterns of **1** below and above the *T*_c_. The ^13^C CSA patterns were extracted from the 2D SUPER^27^ spectra of **1** (see Figures S9 and S10, Supporting Information).

We thus resort to solid‐state ^2^H NMR of sample **1‐d_2_** in which the —NH_2_ group of the BCEA cation is deuterated (**Figure** **4**). Compared with ^13^C CSA analysis, a good advantage of ^2^H NMR lies on that the electric field gradient tensor of ^2^H in a X−^2^H bond is, to a good approximation, axially symmetric (*η*_Q_ = 0) and the *z*‐axis of its principal axis system points approximately along the direction of the X−^2^H bond.^28^, ^29^ Below the *T*_c_, the ^2^H wide line pattern shows a characteristic Pake lineshape with a width of more than 250 kHz, indicating an absence of motion of BCEA cations in the crystal. Above the *T*_c_, the pattern maintains the characteristic Pake lineshape, but the width is reduced by a factor of 0.45. A simulation of the pattern is based on a motional model that the BCEA cation rotates along the long axis of itself (Figure 4). The simulated patterns (gray) are well matched with the experimental ones (black). Note that the ^2^H NMR results only provide the angle between the rotation axis and the two D—N bonds. According to the chemical nature of BCEA cation, four different rotation axes are possible (Figure S12, Supporting Information). To find out the exact rotation axis, we combined the results from the ^2^H NMR and the ^13^C CSA lineshape analysis. This yields two rotation axes (the red and green arrows in Figure 4), which locate in the vertical plane of the angle‐bisecting plane of the D—N—D bond angle. More details on the determination of the rotation axes can be found in Figure S12, Supporting Information.

**Figure 4:**
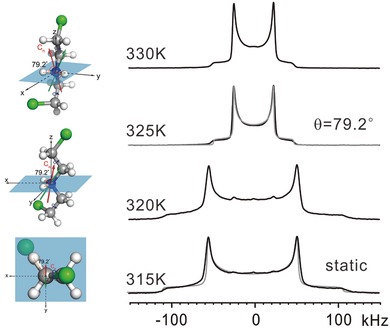
Solid‐state ^2^H NMR spectra of **1‐d_2_** below and above the *T*_c_. The grey lines are the simulated patterns. In the simulation, the electric field gradient tensor of ^2^H is assumed to be axially symmetric (*η*_Q_ = 0) and the *z*‐axis of its principal axis system points along the direction of the N—D bond. Below the *T*_c_, BCEA cations are assume to be “static.” Above the *T*_c_, BCEA cations exhibit an axial rotation. The rotational axes of BCEA cation are in the vertical plane of the angle‐bisecting plane of the D—N—D bond angle and inclined to the two D—N bonds at the same angle of *θ* = 79.2°. The simulated patterns based on this model fit well with the experimental ones. An anti–gauche conformer of the BCEA cation is used as the model.

In summary, an organic salt bis(2‐chloroethyl)amine hydrochloride was found to show a sharp switchable dielectric constant at 320 K. The switching property originates from the dynamic changes of the (2‐chloroethyl)ammonium cation between frozen and motional states, corresponding to a structural phase transition. This simple compound can be potentially used as a molecule‐based switchable electrical and electronic material. It may be also a good model with a close relation to molecular ferroelectrics, molecular rotors, and complicated systems with structural heterogeneity such as liquid crystals and polymers in which motions of chains and polar groups and rich interactions determine their micro/macroscopic properties.

## Supporting information

As a service to our authors and readers, this journal provides supporting information supplied by the authors. Such materials are peer reviewed and may be re‐organized for online delivery, but are not copy‐edited or typeset. Technical support issues arising from supporting information (other than missing files) should be addressed to the authors.

Supplementary
