# Supplementary material for: Switching Dielectric Constant Near Room Temperature in a Molecular Crystal
Source: Adv Sci (Weinh). 2015 Apr 14;2(5):1500029. doi: 10.1002/advs.201500029 (PMC5115362; doi:10.1002/advs.201500029)
Supplement: Supplementary file 1 — Supplementary [file ADVS-2-0q-s001.pdf]

Copyright WILEY-VCH Verlag GmbH & Co. KGaA, 69469 Weinheim, Germany, 2015.

Supporting Information

for *Adv. Sci.*, DOI: 10.1002/advs.201500029

Switching Dielectric Constant Near Room Temperature in a Molecular Crystal

Xiu-Dan Shao, Xi Zhang, Chao Shi, Ye-Feng Yao,* and Wen Zhang*

DOI: 10.1002/advs.201500029
Article type: Communication

Switching Dielectric Constant near Room Temperature in a Molecular Crystal

Xiu-Dan Shao, Xi Zhang, Chao Shi, Ye-Feng Yao, and Wen Zhang**

Xiu-Dan Shao, Chao Shi, Prof. Wen Zhang
Ordered Matter Science Research Center, Southeast University, Nanjing 211189, Jiangsu, China
E-mail: zhangwen@seu.edu.cn
Xi Zhang, Prof. Ye-Feng Yao
Department of Physics & Shanghai Key Laboratory of Magnetic Resonance, East China Normal University, North Zhongshan Road 3663, Shanghai 200062, China
E-mail: yfyao@phy.ecnu.edu.cn

Experimental Section
Sample: Compound **1** is commercially available and recrystallized as colorless crystals from ethanol for further uses.
Measurement: Infrared spectra were taken on a Bruker Vector 22 spectrophotometer as KBr pellets in the 4000−400 cm^{-1} region. Raman spectra were taken using a Horiba Jobin Yvon HR800 spectrometer system with a 488 nm laser line from an air cooled Ar-ion laser. Differential scanning calorimetry (DSC) measurements were performed on a PerkinElmer Diamond DSC under nitrogen at atmospheric pressure with a heating/cooling rate of 5 K min^{-1}. Powder X-ray diffraction (PXRD) was measured on a Rigaku SmartLab X-ray diffraction instrument at various temperatures and beamline BL14B at Shanghai Synchrotron Radiation Facility. For dielectric measurement the crystalline-powdered samples were used in the form of discs. Silver conduction paste was deposited on the surfaces as the electrodes. Dielectric constant was measured with a Tonghui TH2828A impedance analyser over the frequency range of 500 Hz and 1 MHz at a heating/cooling rate of 5 K min^{-1} from 290 to 383 K.
Solid-state NMR experiments: The wide line ^2H NMR was performed on a Bruker Avance III 300 spectrometer operating at 46.07 MHz for ^2H. A Bruker two-channel static PE probe with a homemade 2.5mm coil was used to record the ^2H spectra. The ^2H spectra were acquired using the solid echo sequence ($90^o - \tau - 90^o - \tau -$ acquire). The ^2H pulse width is 2 μs at a RF field strength of $\gamma B_1/2\pi$ = 125 kHz. Below 200 K, the used refocusing delay τ was ~30 μs. Above 200 K, the refocusing delay τ of 20 μs was used. The ^2H patterns were simulated via the weblab (http://weblab.mpip-mainz.mpg.de/weblab/weblab.html). In the simulation, the electric field gradient tensor of ^2H is assumed to be axially symmetric ($\eta_Q = 0$) and the *z*-axis of its principal axis system points along the direction of the N−^{2}H bond. After the transition, BCEA molecules rotate. The rotational axis is inclined to the two D-N bonds at the same angle of θ.
The solid-state NMR experiments were performed with Bruker Avance III spectrometer operating at 400 MHz ^1H Larmor-frequency. A 4mm MAS double-resonance probe was used in the experiments. The 90° pulse length in the experiments varied between 2.5 and 3 μs on both channels, corresponding to $\omega/2\pi$ = 83−100 kHz. The TPPM schemes were applied for dipolar decoupling with the decoupling frequency of $\omega/2\pi$ = 83−100 kHz. Ramped-CP was used for the experiments with cross-polarization step. SUPER (Separation of Undistorted Powder patterns by Effortless Recoupling)[26] experiment was used to obtain undistorted ^{13}C CSA patterns of **1** under MAS. In the CSA recoupling experiments, the spinning speed of rotor was set at 3 kHz. In the ^{13}C CP/MAS experiments, the spinning speed of rotor was set at 10 kHz. The ^{13}C chemical shifts were determined from the carbonyl carbon signal (δ = 176.0 ppm) of glycine relative to tetramethylsilane (TMS). The temperature of the bearing gas was varied for the temperature dependent experiments.
Structure determination: Variable-temperature X-ray single-crystal diffraction data were collected a Rigaku Saturn 924 diffractometer equipped with Rigaku low-temperature gas spray cooler device, by using Mo-Kα (λ = 0.71075 Å) radiation from a graphite monochromator. The crystal temperature was stable to within 2−5 K. Data processing including empirical absorption correction was performed using the CrystalClear software package. The structures were solved by direct methods and successive Fourier synthesis and then refined by full-matrix least-squares refinements on F^2. All non-hydrogen atoms were refined anisotropically and the positions of all hydrogen atoms were generated geometrically. Summary of crystallographic data, selected bond lengths and bond angles and details of hydrogen-bonding interactions for **1** are given in Table S1-3.

CCDC 1036432−1036444 contain the supplementary crystallographic data for this paper. These data can be obtained free of charge from The Cambridge Crystallographic Data Centre via www.ccdc.cam.ac.uk/data_request/cif.

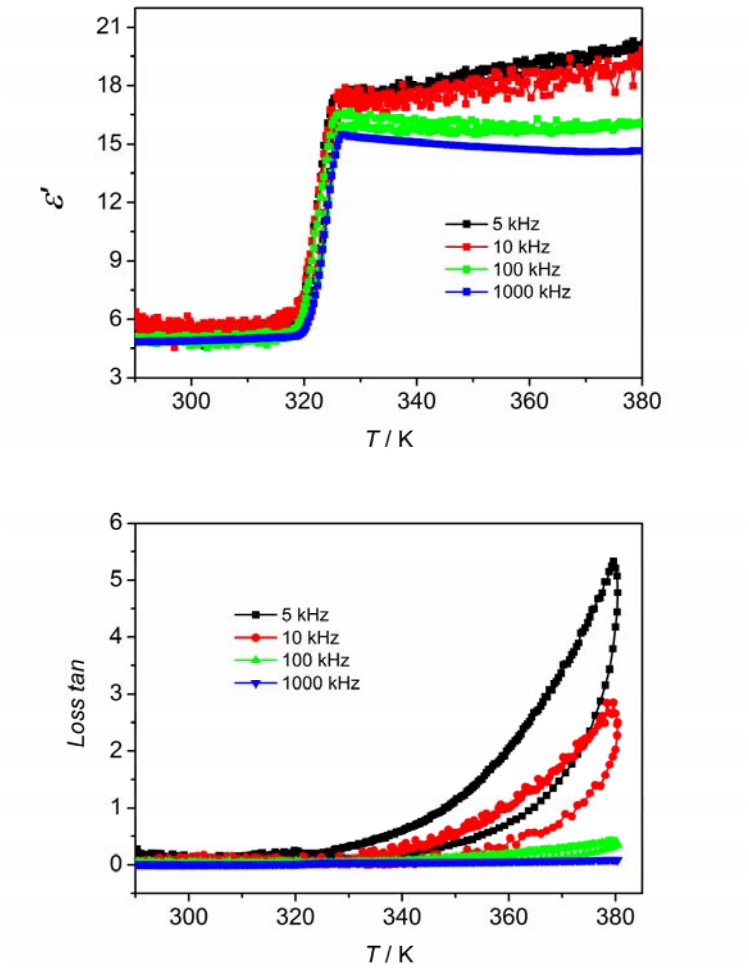

Figure S1. Temperature dependence of the real part of dielectric constant (above) and loss tangent (below) of **1** at different frequencies. For the loss tangent at 310 K, the values are 0.10, 0.05, 0.02 and 0.01 for 5, 10, 100 and 1000 kHz, respectively.

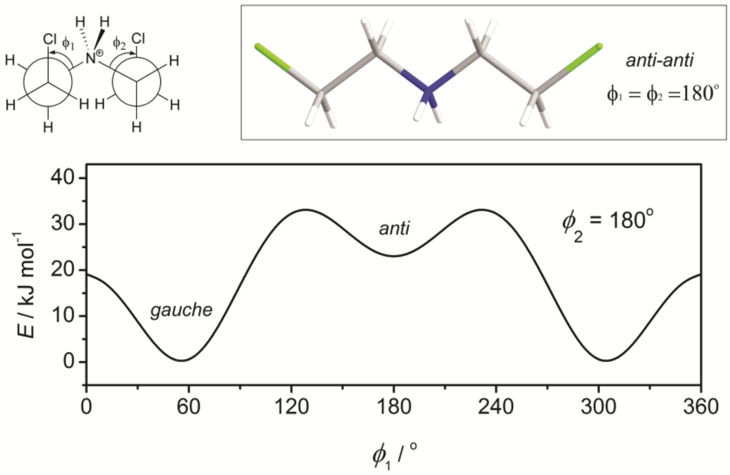

Figure S2. Illustrative potential energy of free BCEA cation in gas phase as a function of the dihedral angle ϕ_1 (Cl−C−C−N).

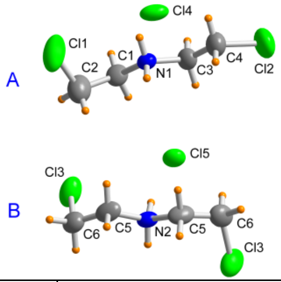			
component	A	B	Cl4+Cl5
all contacts	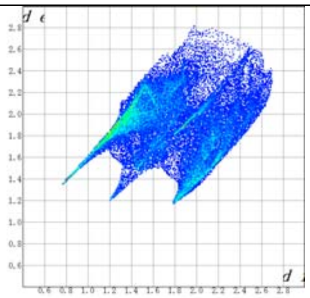	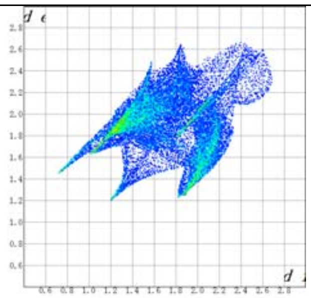	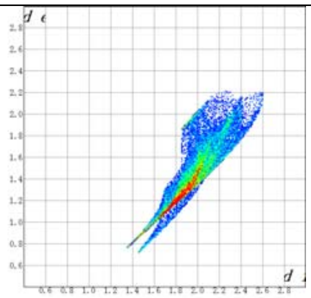
$H_i \cdots H_e$	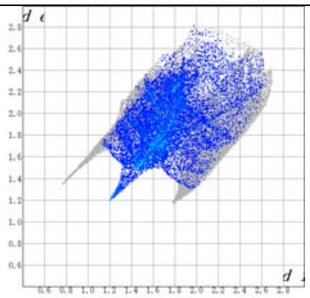	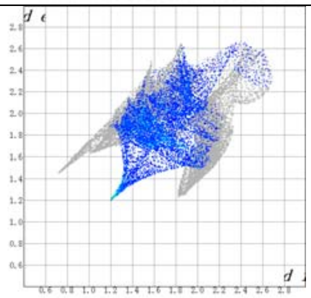	
$H_i \cdots Cl_e$	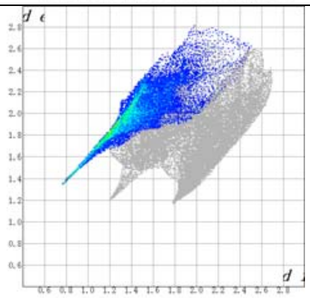	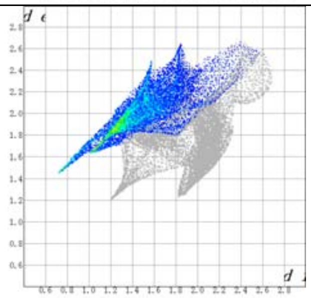	
$Cl_i \cdots H_e$	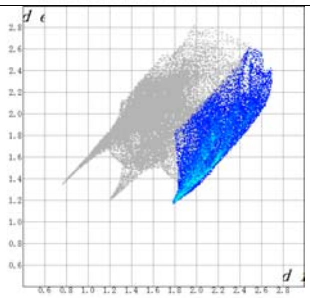	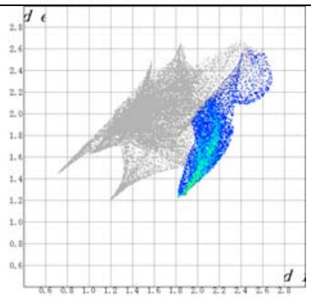	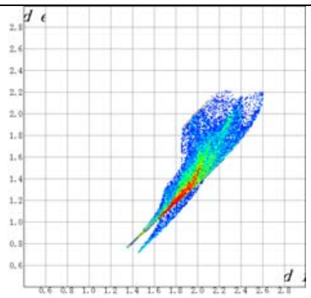

$Cl_i \cdots Cl_e$	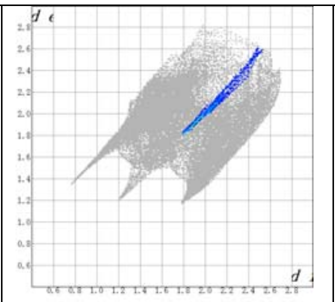	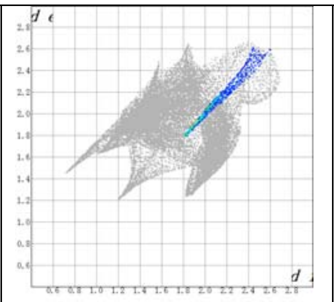	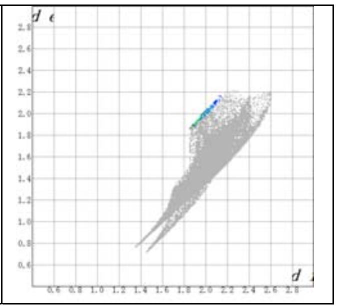

Figure S3. 2D fingerprint plots of the components in **1**, showing atomic contacts to the Hirshfeld surface. In the plot, the d_i and d_e denote the distances from the surface to the nearest atom interior and exterior to the surface, respctively.

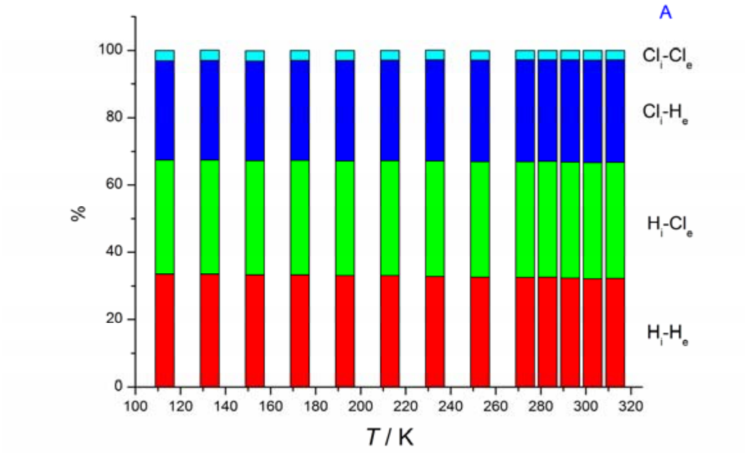

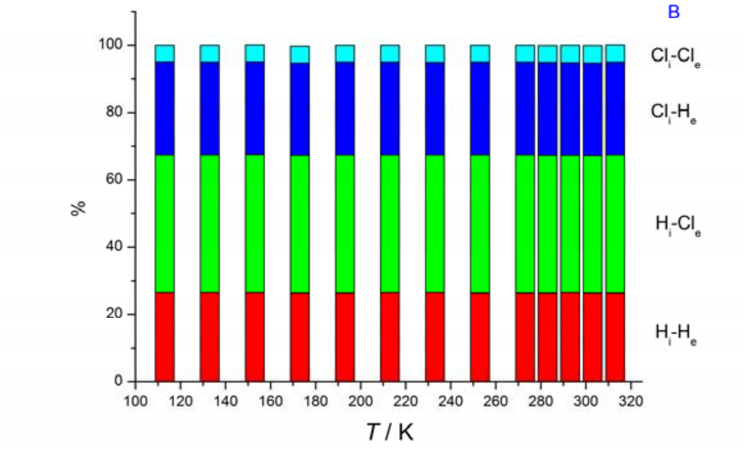

Figure S4. Percentage contributions of atomic contacts to the Hirshfeld surfaces of A and B cations in **1**. The *i* and *e* denote atoms interior and exterior to the surface, respectively.

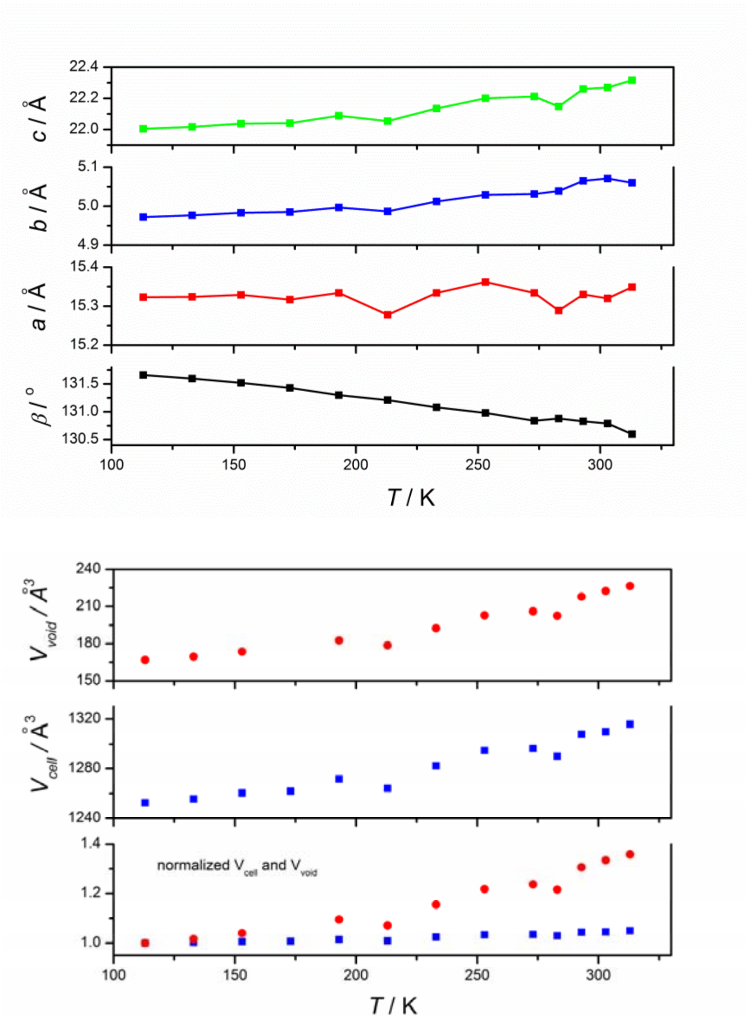

Figure S5. (above) Changes of cell parameters of **1**. (below) The void volumes are calculated based on the isovalue of 0.002 au, corresponds to a smoothed van der Waals surface.

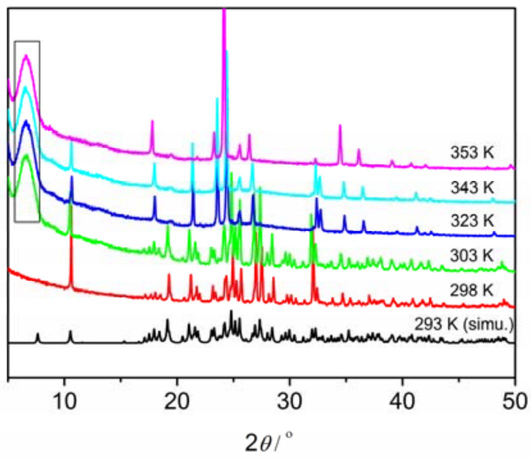

Figure S6. Variable temprature PXRD patterns of **1**. The broad peaks at 6.5° are from the heating stage.

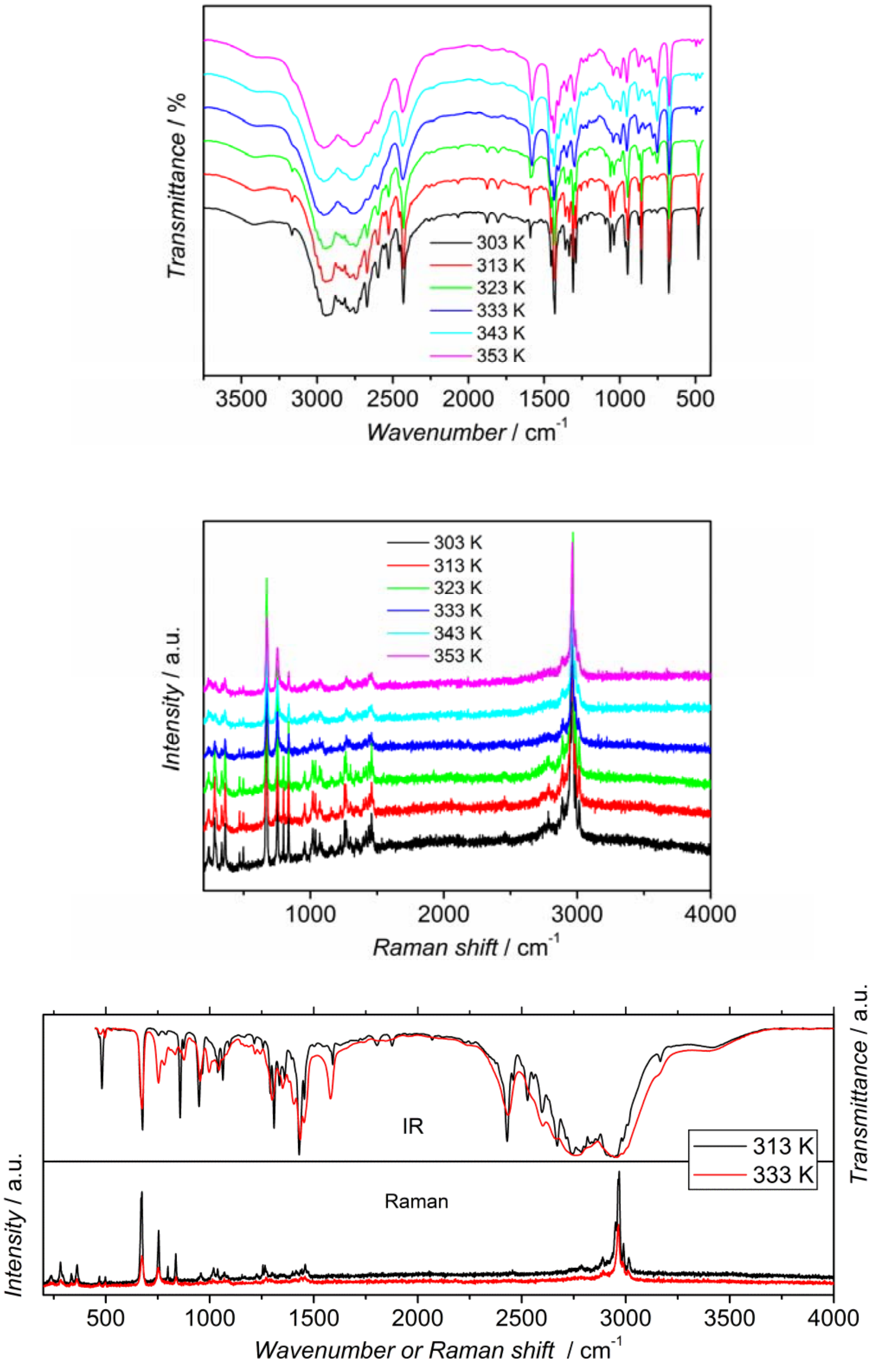

Figure S7. Variable-temperature IR (above) and Raman (middle) spectra of **1** measured in the temperature range 303−353 K. (below) Comparison of the curves at two selected temperatures at 313 K and 333 K, respectively.

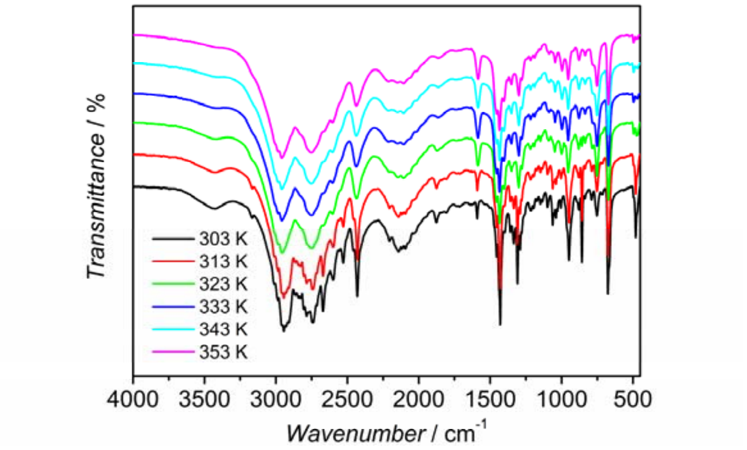

Figure S8. Variable-temperature IR (above) and Raman (below) spectra of *N*-deuterated **1** measured in the temperature range 303−353 K.

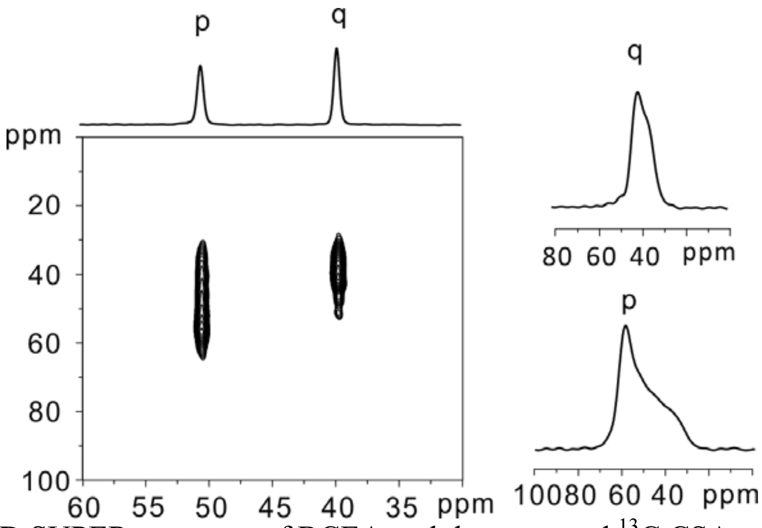

Figure S9. The 2D SUPER spectrum of BCEA and the extracted ^{13}C CSA patterns. The experimental temperature is 340 K.

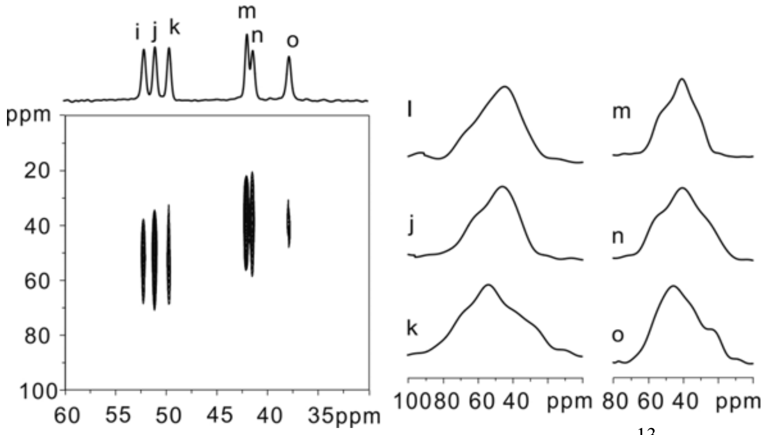

Figure S10. The 2D SUPER spectrum of BCEA and the extracted ^{13}C CSA patterns. The experimental temperature is 310 K.

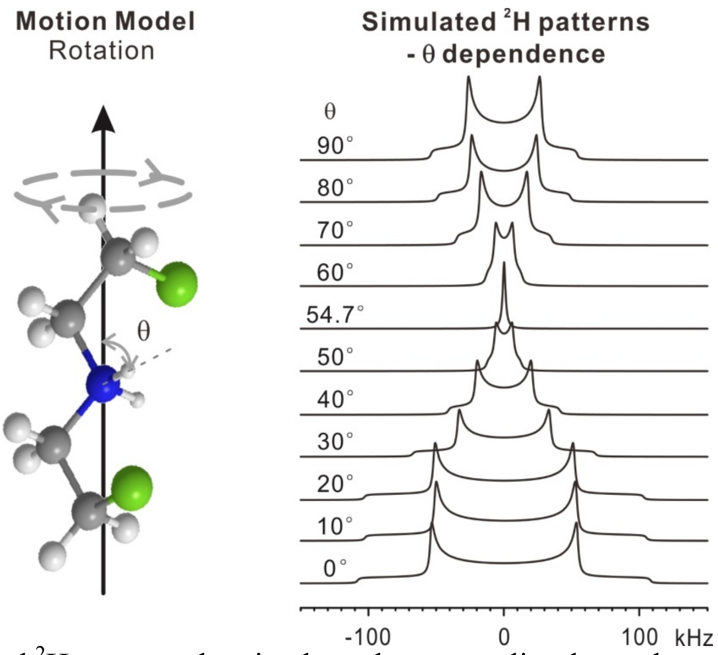

Figure S11. Simulated ^{2}H patterns showing how the pattern lineshape changes with the θ angle.

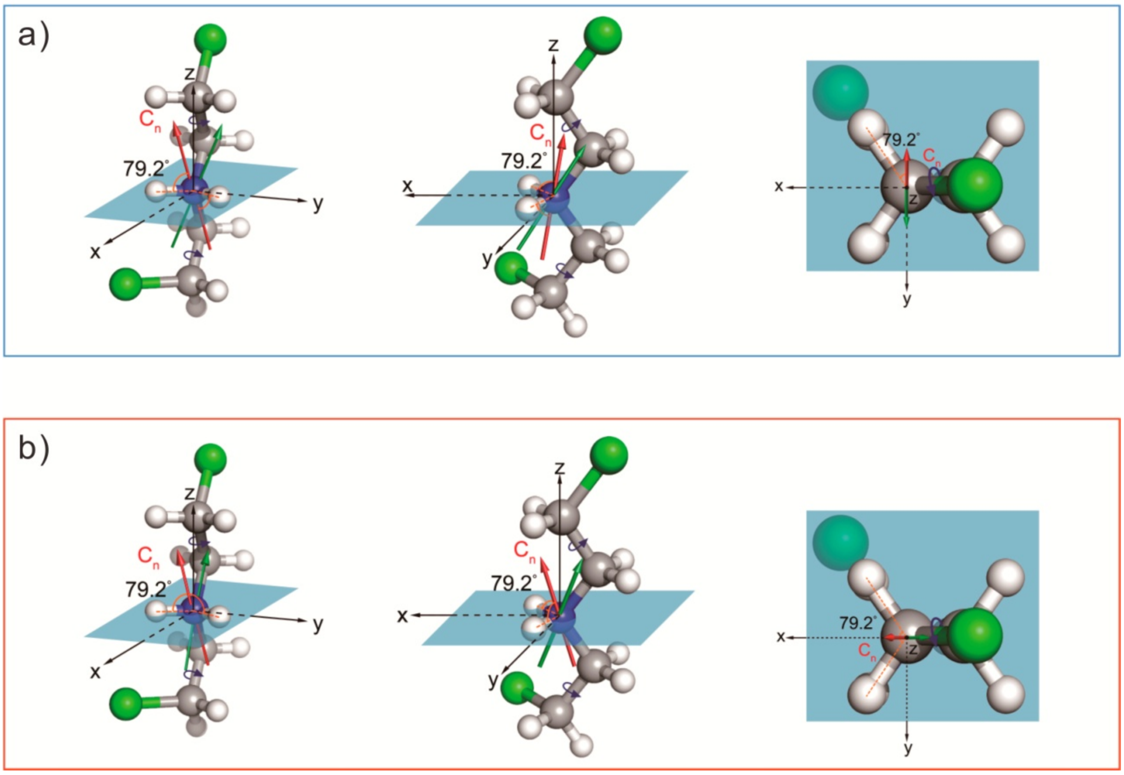

Figure S12. Cartoon pictures of rotating BCEA cations viewed along different directions. These pictures exhibit four rotational axes of BCEA, which are all inclined to two D−N bonds at the same angle of $\theta = 79.2^{o}$. The two axes in (a) are in the vertical plane of the angle-bisecting plane of the D−N−D bond angle, whereas the two axes in (b) are in the angle-bisecting plane of the D−N−D bond angle. Combinition of the ^{13}C CSA analysis and the ^{2}H

NMR indicates that BCEA molecules in all likelihood rotate along the two axes in (a) above the T_c. An *anti*-*gauche* conformer of the BCEA cation is used as the model.

To determine the rotational axes of BCEA, the following points/facts are considered:
1. In the ^{2}H patterns, only one Pake pattern is observed. This indicates that the rotational axes must be inclined to the two D−N bonds at the same angle. This yields the four rotational axes shown in Figure S12.
2. Above the T_c, the ^{13}C CSA power pattern of the CH_2 group shows only one lineshape, and so does the CH_2Cl group. This indicates that the CH_2 and CH_2Cl groups above and under the D−N−D plane are similarly modulated by the axial rotation. This excludes the two rotational axes in Figure S12b, which will most likely result in different modulations on the ^{13}C CSA tensors of the CH_2 and CH_2Cl groups above and under the D−N−D plane.

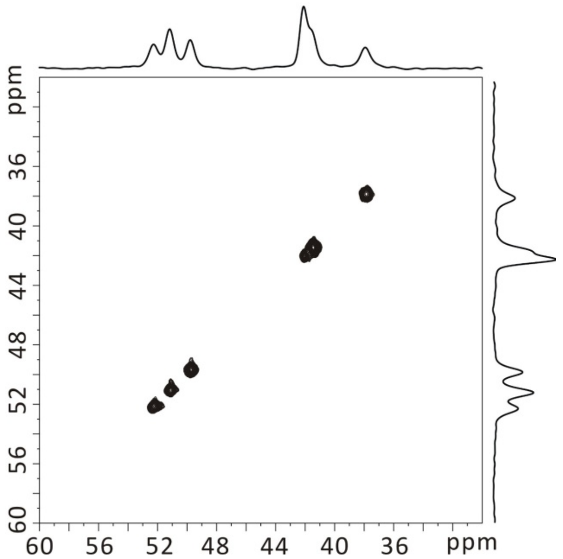

Figure S13. ^{13}C 2D exchange spectrum of BCEA measured at 310 K. The exchange time is 1 s.

Table S1 Crystallographic data and structural refinement details for **1** at 253 K.

Formula	$C_4H_{10}Cl_3N$
Formula weight	178.48
Crystal size / mm	0.28×0.30×0.34
T / K	253(2)
Crystal system	monoclinic
Space group	*P2/c*
a / Å	15.36(2)
b / Å	5.029(4)
c / Å	22.20(1)
α / °	90
β /°	130.98(3)
γ /°	90
V / $Å^3$	1295(2)
Z	6
D_{calc} / g·cm^{-3}	1.373
μ / mm^{-1}	0.976
F(000)	552
θ range / °	3.51−27.40
Reflns collected	13336
Independent reflns (R_{int})	2958 (0.0502)
no. parameters	110
R_1[a], wR_2[b] [I>2σ(I)]	0.0651, 0.1568
R_1, wR_2 [all data]	0.0863, 0.1700
GOF	1.076
$\Delta\rho$[c] / e·Å^{-3}	1.354, −0.658

[a] $R_1 = \Sigma||F_o| - |F_c||/\Sigma|F_o|$. [b] $wR_2 = [\Sigma w(F_o^2 - F_c^2)^2/\Sigma w(F_o^2)^2]^{1/2}$. [c] Maximum and minimum residual electron density.

Table S2 Bond lengths (Å) and torsion angles (°) for **1** at 253 K.

C(2)−Cl(1)	1.756(5)	N(1)−C(1)−C(2)−Cl(1)	−62.0(4)
C(4)−Cl(2)	1.790(4)	N(1)−C(3)−C(4)−Cl(2)	−177.7(3)
C(6)−Cl(3)	1.797(4)	N(2)−C(5)−C(6)−Cl(3)	65.1(4)
		C(2)−C(1)−N(1)−C(3)	−162.4(3)
		C(4)−C(3)−N(1)−C(1)	−179.1(3)
		C(6)−C(5)−N(2)−C(5)#1	170.6(4)

Symmetry transformations used to generate equivalent atoms: #1 −x+1, y, −z+1/2.

Table S3 Hydrogen bonds (Å, °) for **1** at 253 K.

D−H···*A*	*D*−H	H···*A*	*D*···*A*	*D*−H···*A*
N(1)−H(1A)···Cl(4)	0.90	2.25	3.128(3)	166.0
N(1)−H(1B)···Cl(5)	0.90	2.22	3.109(3)	171.4
N(2)−H(2C)···Cl(5)#2	0.90	2.27	3.155(3)	166.0
N(2)−H(2D)···Cl(5)#3	0.90	2.27	3.155(3)	166.0

Symmetry transformations used to generate equivalent atoms: (ii) x, y+1, z; (iii) −x+1, y+1, −z+1/2.
